# Fluorescent Affibody Peptide Penetration in Glioma Margin Is Superior to Full Antibody

**DOI:** 10.1371/journal.pone.0060390

**Published:** 2013-04-12

**Authors:** Kristian Sexton, Kenneth Tichauer, Kimberley S. Samkoe, Jason Gunn, P. Jack Hoopes, Brian W. Pogue

**Affiliations:** 1 Thayer School of Engineering at Dartmouth College, Hanover, New Hampshire, United States of America; 2 Department of Surgery, Geisel School of Medicine, Dartmouth College, Hanover, New Hampshire, United States of America; The Ohio State University Medical Center, United States of America

## Abstract

**Object:**

Fluorescence imaging has the potential to significantly improve neurosurgical resection of oncologic lesions through improved differentiation between normal and cancerous tissue at the tumor margins. In order to successfully mark glioma tissue a fluorescent tracer must have the ability to penetrate through the blood brain barrier (BBB) and provide delineation in the tumor periphery where heterogeneously intact BBB may exist. In this study it was hypothesized that, due to its smaller size, fluorescently labeled anti-EGFR Affibody protein (∼7 kDa) would provide a more clear delineation of the tumor margin than would fluorescently labeled cetuximab, a full antibody (∼150 kDa) to the epidermal growth factor receptor (EGFR).

**Methods:**

Cetuximab and anti-EGFR targeted Affibody were conjugated to two different fluorescent dyes (both emitting in the near-infrared) and injected intravenously into 6 athymic mice which were inoculated orthotopically with green fluorescent protein (GFP) expressing human U251 glioma cells. Each mouse was sacrificed at 1-h post injection, at which time brains were removed, snap frozen, sectioned and quantitatively analyzed for fluorescence distribution.

**Results:**

*Ex vivo* analysis showed on average, nearly equal concentrations of cetuximab and Affibody within the tumor (on average Affibody made up 49±6% of injected protein), however, the cetuximab was more confined to the center of the tumor with Affibody showing significantly higher concentrations at the tumor periphery (on average Affibody made up 72±15% of injected protein in the outer 50 um of the tumor). Further *ex vivo* analysis of detection studies showed that the Affibody provided superior discrimination for differentiation of tumor from surrounding normal brain.

**Conclusions:**

The present study indicates that fluorescently labeled anti-EGFR Affibody can provide significantly better delineation of tumor margins than a fluorescently labeled anti-EGFR antibody and shows considerable potential for guiding margin detection during neurosurgery.

## Introduction

Fluorescence imaging technology may have its greatest clinical potential in the rapidly expanding field of fluorescence-guided neurosurgery. [1]–[6] The key to fluorescence guided surgical oncology is the ability to create specific contrast between normal and glioma tissue. This, together with a fluorescence-enabled surgical microscope, allows removal of molecular-defined portion of the tumor while at the same time minimizing removal of normal brain. The prognosis of patients suffering from malignant gliomas has been linked to the completeness of tumor removal and the ability to selectively mark tumor tissue with fluorescence has already shown promise to improve outcomes through reduced margins in surgical resection. [7]–[9] In this study, two potential fluorescent cellular receptor targeting agents of different size are compared in terms of their ability to mark the outer regions of glioma tumors. The hypothesis tested here is that smaller binding agents would better define the infiltrative edge of the tumor.

Fluorescent contrast enhancement of malignant gliomas was first reported on in 1948 by Moore et al. where an injection of fluorescein was preferentially taken up by the tumor compared to the normal brain tissue as a result of the tumor’s disrupted blood brain barrier (BBB). [10] While the use of fluorescein continues to be examined today, [11] the preponderance of research in the area of fluorescence guided surgery has focused on the administration of 5-aminolevulinic acid (5-ALA), a natural precursor of protoporphyrin IX (PpIX) in the heme biosynthesis pathway. [12], [13] PpIX is selectively synthesized in high grade glioma, with normal brain having extremely low concentrations [14], [15] and the resulting fluorescence contrast has been used to reduce margins in surgical resection. [8], [16] This approach, however, is not without its limitations and one of the primary is that its maximal useful signal seems to be restricted to high grade gliomas [17], [18].

One promising yet little explored method for differentiating tumor from normal brain tissue in surgical resection is the administration of fluorescently labeled targeted proteins. An important advantage of this over the simple administration of untargeted fluorescent tracers such as fluorescein or indocyanine green [19] is that it could provide specificity through the targeting of overexpressed glioma cell surface receptors. Contrast with this approach is governed largely by receptor-ligand affinity and receptor density rather than cellular metabolism as is the case in PpIX approaches [12], [14], [15] and as a result targeted fluorescence imaging will not suffer from the problem of reduced PpIX production encountered in low-grade gliomas. However, this approach is not without its own unique problems, one of which is the difficulty in establishing receptor status prior to any initial surgery. It must also be pointed out that the tumor used in the present study, U251, is fact a high grade glioma and any specific problems associated with low grade gliomas and the use of targeted fluorescent probes will not be seen in this study. Another concern with the approach used is that the dye-protein conjugates, which are much larger than 5-ALA or fluorescein, may be too large to adequately penetrate tumor areas with a partially intact BBB and we must keep in mind that breakdown of the BBB is less pronounced in low grade gliomas.

The BBB generally limits delivery of imaging agents to the normal brain, but in tumors this is typically compromised to an extent that allows for sufficient contrast in imaging of the bulk tumor. One area of concern, however, is that BBB breakdown is often incomplete, particularly in newly formed areas of growth including micro-invasive regions. [20]–[22] The result is that exogenously administered agents tend to accumulate in the tumor interior where breakdown of the BBB is most complete, but not necessarily in the infiltrative edges where the BBB more closely resembles that of the normal brain. [21]–[23] Delivery to these areas is of the utmost importance if these methods are to achieve clinical success.

As a preliminary investigation into this area, the present study compares delivery of two promising targeted proteins – a full antibody and an Affibody, which have substantial differences in size and target affinity to orthotopic human gliomas grown in a murine model. The proteins are targeted to epidermal growth factor receptor (EGFR), a cell-surface receptor that is over-expressed in many human gliomas [24], [25] However, it must be noted that EGFR is rarely over-expressed in low grade diffuse gliomas and this prevents it from being an optimal target for marking this type of tumor. While low grade diffuse gliomas present the most difficult category to treat they are not the only type of glioma that could benefit from improvements in fluorescence contrast. High grade gliomas for which PpIX fluorescence is most well suited may also benefit from the use of targeted fluorescent probes possibly as a tool to be used in combination with PpIX. Additionally, as more promising targets on low grade gliomas are elucidated, the present work may help to inform the development and testing of targeted proteins for these receptors. Integrin α_v_β_3_ is one such cell surface receptor that has already been shown to be overexpressed on low grade gliomas. [26] In the present study, the penetration of the two proteins to the center and periphery of tumors was examined by fluorescent imaging of *ex vivo* brain slices collected from mice 1 h after intravenous injection of the fluorescently labeled proteins.

## Materials and Methods

### 2.1 Protein Labeling

The smaller of the two proteins used to target EGFR, anti-EGFR Affibody (Affibody AB, Solna, Sweden), was diluted with phosphate buffered saline (PBS) at pH 7.5 to achieve a concentration of 1 mg/ml. As per the manufacturer’s recommendations, the Affibody molecules were reduced by adding dithiothreitol (DTT, mM) and incubated on a magnetic stirrer for two hours at room temperature. Excess DTT was removed by passage through a polyachrylamide 6000 desalting column (Thermo Scientific, Rockford, IL). Recovered protein was concentrated in a centrifuge using a 6 kDa molecular weight cutoff (MWCO) column (GE Vivaspin 2,Pittsburgh, PA). At this point the Affibody was ready for binding with a fluorophore. The fluorophore, IRDye 800CW maleimide (LI-COR Biosciences, Lincoln, Nebraska), was suspended in pure water at approximately 2 mg/ml, and was added to the protein solution to achieve a 2.5 molar excess of dye to protein as recommended by LI-COR. The Affibody-IRDye 800CW solution was then incubated on a magnetic stirrer for approximately two hours at room temperature, excess dye was removed by passage through a desalting column, and concentrated in the centrifuge using a 6 kDa MWCO column. A dilution made from the concentrated labeled Affibody solution was examined in a UV-Vis spectrophotometer (Cary 50 BIO UV-Visable spectrophotometer, Varian, Palo Alto, CA) to record the absorption spectrum from 220–800 nm. Protein concentrations and dye-to-protein ratios were determined using absorption values at 280 nm and 780 nm as described by LI-COR. All labeled Affibody solutions yielded dye-to-protein ratios between 0.65 and 0.73.

The larger of the two EGFR-targeted proteins, cetuximab (ImClone Systems, Inc, NewYork, NY) was labeled with the fluorophore, IRDye 680RD NHS ester (LI-COR). 2 mg/ml of the cetuximab was added to a 5-mg/ml solution of the fluorophore suspended in DMSO to achieve a 3 molar excess of dye to protein as recommended by LI-COR. The cetuximab-IRDye 680RD solution was incubated on a magnetic stirrer for approximately two hours at room temperature. Excess dye was removed by passage through a polyachrylamide 6000 desalting column. The labeled protein solution was then concentrated in a centrifuge within a 50-kDa MWCO column (GE Vivaspin 2, Pittsburgh, PA). The absorption spectrum of the labeled cetuximab was recorded from 220–700 nm using the UV-Vis spectrophotometer to measure concentrations and dye-to-protein ratios (dye-to-protein ratios were between 1.74 and 1.87).

For all animal injections, cetuximab-IRDye680RD and Affibody-IRDye800CW are mixed together and injected simultaneously. In order to rule out the possibility of binding between the two proteins, the Octet Red 96 (forteBIO, Menlo Park, CA) which uses biolayer interferometry to identify molecular binding, was employed. Cetuximab-IRDye680RD was captured on Protein A. Affibody-IRDye800CW was diluted to the same concentration used for all injections (0.05 *u*M) and allowed to mix with the immobilized cetuximab_IRDye680RD for approximately thirty minutes. No binding between the proteins was seen.

### 2.2 Animal Models

All animals were used in accordance with an approved protocol and the policies of the Institutional Animal Care and Use Committee (IACUC) at Dartmouth College. Twenty-six, six-week-old female nude mice were obtained from Charles River Laboratories (Wilmington, MA) and randomly separated into three experimental groups. Fourteen animals were used in a plasma excretion study and the remaining twelve animals were inoculated with orthotopic implantations of human glioma cell line, and either injected with a mixture of the two EGFR-targeted tracers (*n* = 6) or used as naive controls (*n* = 6).

A green fluorescent protein (GFP) expressing human neuronal glioblastoma cell line, U251-GFP (supplied from Dr. Mark Israel, Norris Cotton Cancer Center, Dartmouth-Hitchcock Medical Center and transfected with GFP in our lab), was selected for implantation because it is a cell line known to express moderate levels of the targeted receptor, EGFR. [27], [28] Implantations were carried out under anesthesia (90∶10 mg/kg ketamine:xylazine). A small incision was made in the scalp, exposing the landmarks on the skull, and a 1-mm rotary drill was used to create an access to the brain, 2 mm behind the bregma and 2 mm to the left of the midline. 5×10^5^ U251-GFP cells were injected at a 2 mm depth into the left cerebral hemisphere of the mice using in 5 uL of phosphate-buffered saline (PBS) using a Hamilton syringe (Hamilton Company, Reno, NV), guided by a non-digital stereotaxic frame fitted with tubing to allow isoflurane anesthesia (Stoelting Co, Wood Dale, IL). Based on the atlas of the adult mouse brain this places the tumor in the area of the dorsal nucleus of lateral geniculate body. [29] The cells were injected over a 5-minute period, after which the needle was slowly retracted from the brain. Bone wax (Ethicon, Inc, Piscataway, NJ) was used to close the hole in the skull while the incision in the scalp was closed using Vetbond (J.A. Webster, Inc, Sterling, MA) [30]–[32].

One week following tumor implantation, mice were placed on a non-fluorescent diet (Purified Mouse Diet, CAT NO. 904606, from MP Biomedicals, LLC, Illkirch, France) and tumors were allowed to grow for two further weeks before carrying out fluorescent tracer experiments.

### 2.3 Plasma Clearance

The plasma excretion rates of cetuximab-IRDye680RD and anti-EGFR Affibody-IRDye800CW were determined by monitoring the fluorescence in mouse blood for 24 h following intravenous injection of a mixture containing between 0.1 and 0.75 nmols of each protein tracers. At selected time points (all mice at 1 min and then at three additional time points within 24 h), approximately 150 µL of blood was collected via a submandibular bleeding technique using a 5 mm lancet (Goldenrod; MEDIpoint, Mineola, NY) into a vial previously rinsed with Heparin (Hospira, Lakeforest, IL). Three mice had pre-injection blood samples collected to enable determination of the autofluorescence spectrum. The blood samples were centrifuged and the resulting plasma layer removed for analysis on a fluorimeter (Fluoromax-3, Horiba Jobin Yvon, Edison, NJ). Cetuximab-IRDye680RD was analyzed using an excitation of 620 nm over an emission range of 650–800 nm while anti-EGFR Affibody-IRDye800CW was analyzed using an excitation 720 nm over an emission range of 730–900 nm. The baseline of each fluorescence spectra was determined by fitting a fourth degree polynomial and baselines were then subtracted from the spectra. The resulting spectra were then integrated over 10 nm at the fluorescent peaks. Autofluorescence was determined in the same manner using the pre-injection samples and the average integrated signal for autofluorescence was then subtracted from each sample. The 1 min post-injection blood sample was used to normalize fluorescence intensities and the resulting data was then fit to a bi-exponential decay. All calculations were performed using Matlab 2009a (Mathworks, Natick, MA) [33], [34].

### 2.4 Tracer Uptake in Tumor

To determine the relative uptakes of the cetuximab and Affibody based EGFR-targeted tracers in an EGFR-expressing glioma, 0.1 nmol of each tracer was simultaneously injected intravenously into six of the twelve mice implanted with U251-GFP tumors. At 1 h post injection mice were euthanized by cervical dislocation under ketamine-xylazine (90∶10 mg/kg i.p.) anesthesia. The 1- h time point was chosen for its potential future clinical feasibility in guiding surgical resection. Brains were extracted, covered in optimum cutting temperature (OCT) medium (Tissue Tek®, Sakura Finetek USA, Inc., Torrance, CA), snap frozen at −60°C in methylbutane and dry ice, and stored at −80°C until used for sectioning. The six control mice were treated the same as the others except that they were not injected with the cetuximab and Affibody. This provided a means of investigating the level of autofluorescence in tumor and normal brain tissue (*i.e.*, fluorescence in the absence of any injected dyes). One mouse from the group injected with the two proteins failed to grow an observable tumor, leaving five mice in that group.

### 2.5 Tissue Sectioning and Imaging

Tissue sections (10 µm in thickness) were prepared on a cryotome (CM 1850, Leica Microsystems, Richmond, IL), placed on glass slides (Precleaned Gold Seal Rite-on Micro Slides, Gold Seal Products, Portsmouth, NH) and stored at −80°C. Fluorescence from GFP in the frozen sections was imaged on the Typhoon 9410 Variable Mode Imager (GE Healthcare, Milwaukee, WI) at 25 micron resolution (488 nm excitation, emission at 500–540 nm). Comparison of GFP-expression with a sampling of adjacent hematoxylin and eosin (H&E) stained tissue sections confirmed that GFP signal accurately outlined tumor regions. Tissue sections were then scanned on the Odyssey Infrared Imaging System (LI-COR Biosciences) at 21 µm resolution with a gain of 9.0 selected in both the 700 and 800 nm channels. These settings provided adequate signal without saturation, allowing for the quantification of the level of the cetuximab and Affibody tracers. Between thirty and sixty tissue slices were examined from each mouse.

### 2.6 Protein Concentration Quantification

Protein concentrations at the tumor edge, tumor interior and over the whole tumor were quantified from Odyssey fluorescence images using the following equation:
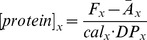
where *F_x_* represents fluorescence of the select region at x = 700 nm or x = 800 nm for the cetuximab and Affibody tracers, respectively; 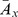
is the average autofluorescent level at 700 or 800 nm measured from the uninjected brain slices in the corresponding region (tumor edge, tumor interior or whole tumor); *DP_x_* is the dye-to-protein ratio of the corresponding tracer; and *cal_x_* is a fluorescence to dye concentration calibration factor for the each tracer. The calibration factors for the two tracers were determined by carrying out serial dilution experiments on Odyssey system.

### 2.7 Image Analysis

The Odyssey fluorescence images were scaled using bicubic interpolation to produce the same 25 µm pixel size recorded using the Typhoon. All three sets of images were then aligned using a combination of manual point selection and automated cross correlation in Matlab R2009a (Mathworks, Natick, MA). Approximately one out of every ten slides was viewed using ImageJ (National Institute of Health) to verify coregistration accuracy.

Tumor segmentation was carried out using a GFP threshold; *i.e.*, all pixels having a GFP signal greater than three standard deviations above the mean of the contralateral region were regarded as tumor and all those below being regarded as normal brain. The outer two pixels or 50 um of each tumor was defined as the tumor edge while the tumor interior was defined as the region more than five pixels or 125 um from the actual tumor edge. Only continuous regions of tumor with areas greater than 0.625 mm^2^ were used in this sub-analysis.

### 2.8 Statistical Analysis

For comparison purposes the means and standard deviations of the signals at both channels were calculated for each examined region as well as for all metrics used. These are reported as: mean ± standard deviation, throughout the manuscript.

Statistical analysis was performed using R version 2.15.1 from the R foundation for statistical computing. Welch Two Sample t-tests were performed to determine statistical significance in the parameters related to differences in signal from the Affibody and cetuximab channels in different regions of the tumor for the five protein injected animals. These parameters included the difference in Affibody protein fraction between the tumor interior and tumor edge as well as the differences in signal decrease from the tumor interior to the tumor edge for the two channels. A Welch Two Sample t-test was also used to determine the statistical significance of the difference in signal between the tumor regions and the contralateral regions in the non-injected control animals at both the 700 and 800 nm channels.

## Results

**Figure 1:**
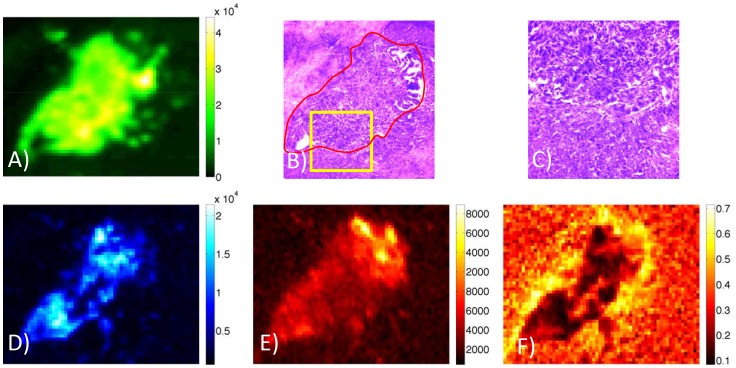
Examination of Affibody and cetuximab distribution over the tumor region. Signal from GFP outlines the tumor (A). H&E stain of the same tissue slice showing the structural differences between the tumor area and adjacent normal tissue at 8 times magnification (B). Tumor is outlined in red and area enclosed in the yellow box is shown at 20 times magnification in (C). Fluorescent signal at cetuximab channel shows significant contrast in much of the tumor, but appears reduced around the edges (D). Fluorescent signal at Affibody channel shows significant contrast in the tumor and over a broader region of the tumor (E). Fraction of signal from the Affibody channel is shown in (F) and demonstrates significant deviation in signal from the two channels at the edge and interior of the tumor.

Fig. 1. shows the GFP outlined tumor, H&E staining of the same tissue section and raw fluorescence at both the Affibody and cetuximab channels. The most striking observation of the Affibody and cetuximab maps is that on average cetuximab appears to be more confined to tumor interiors (Fig. 1D), while the Affibody appears more evenly dispersed throughout the tumor (Fig. 1E). Maps of the percentage of signal from the Affibody channel (Affibody fluorescence/(Affibody fluorescence+cetuximab fluorescence)) reinforced this observation, illustrating a clear increase in Affibody at the tumor margins compared to cetuximab (Fig. 1F).

**Figure 2:**
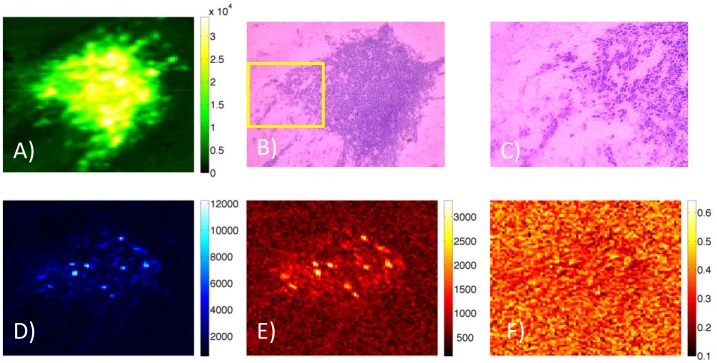
Examination of autofluorescence at both Affibdoy and cetuximab channels over the tumor region Tumor outlined by GFP signal (A). H&E stain of the same tissue slice showing the structural differences between the tumor area and adjacent normal tissue at 8 times magnification (B). Area enclosed in the yellow box is shown at 20 times magnification in (C). Autofluorescence at both the cetuximab channel (D) and the Affibody channel (E) show significant contrast between tumor and non-tumor regions with autofluorescence greatest at the tumor center. No significant change between tumor interior, tumor edge and non-tumor area is seen for the fraction of signal at the Affibody channel (F).

Control mice that received tumor implantations but no protein injections were used to determine background tissue signals. Tumor autofluorescence proved to be significantly higher than the autofluorescence in the brain tissue at both the 700 and 800 nm channels.(*p* = 0.011 and *p* = 0.005, respectively). This is demonstrated by the ability to localize the tumor somewhat based on autofluorescence alone at both channels (Fig. 2D & 2E). Autofluorescence signal was found to be most pronounced at the tumor interiors and lowest at tumor edges, with a fluorescence level of 1203±132 and 610±31 in the tumor margin and 1424±284 and 691±83 in the tumor interior for the 700 and 800 nm channels, respectively. The fraction of signal from the 800 nm channel, which can be contrasted with the Affibody fraction image seen for an injected animal in Fig. 1F, did not show any spatial pattern between tumor interior, tumor edge, or surrounding tissue (Fig. 2F).

**Figure 3:**
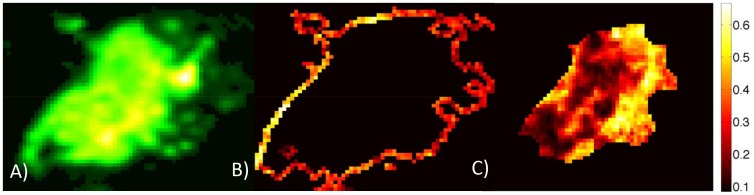
Illustration of procedure used in analysis. Tumor is segmented using GFP signal (A). The outer 50 um edge of the tumor is separated from the rest of the tumor (B) and the inner portion of the tumor at a distance greater than 125 um from the edge is also separated (C). Scale in (B) and (C) show fraction of signal at affibody channel.

**Figure 4:**
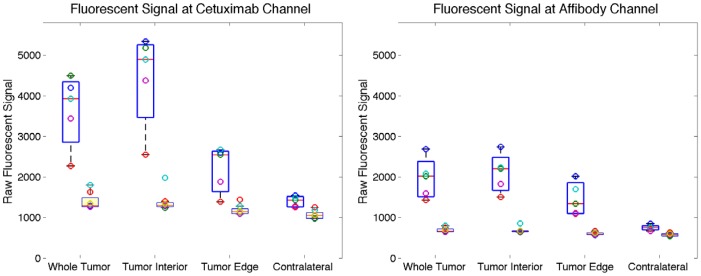
Comparison of raw fluorescent signals. Raw fluorescent signals from various regions shown at both cetuximab channel (left) and Affibody channel (right) using box and whisker plots. Signal from injected animals are offset to the left while those from non-injected control animals are offset to the right with boxes shaded. The central lines are the medians, the edges of the boxes are the 25th and 75th percentiles and individual data points are plotted as open circles.

The apparent spatial differences in Affibody and cetuximab uptake in the tumors (*i.e.*, that the Affibody appeared to penetrate the margins of the tumors better than the cetuximab) were quantified by dividing the signal from each tumor into an edge region and an interior region as described in the Materials and Methods section. A graphic illustration of this delineation is presented in Fig. 3, where both the tumor edge (Fig. 3B) and tumor interior (Fig. 3C) masks are shown alongside the entire segmented tumor (Fig. 3A). Raw signals from these regions as well as from a contralateral region were collected from all five injected animals, as well as the six control animals and are plotted in Fig. 4. This analysis demonstrates that the signal from both tracers decreased from the tumor interior to the tumor edge, however, the decrease was significantly larger for the cetuximab tracer for which signal dropped an average of 50±5% vs. the Affibody tracer for which signal dropped only 31±7% (*p* = 0.002).

**Figure 5:**
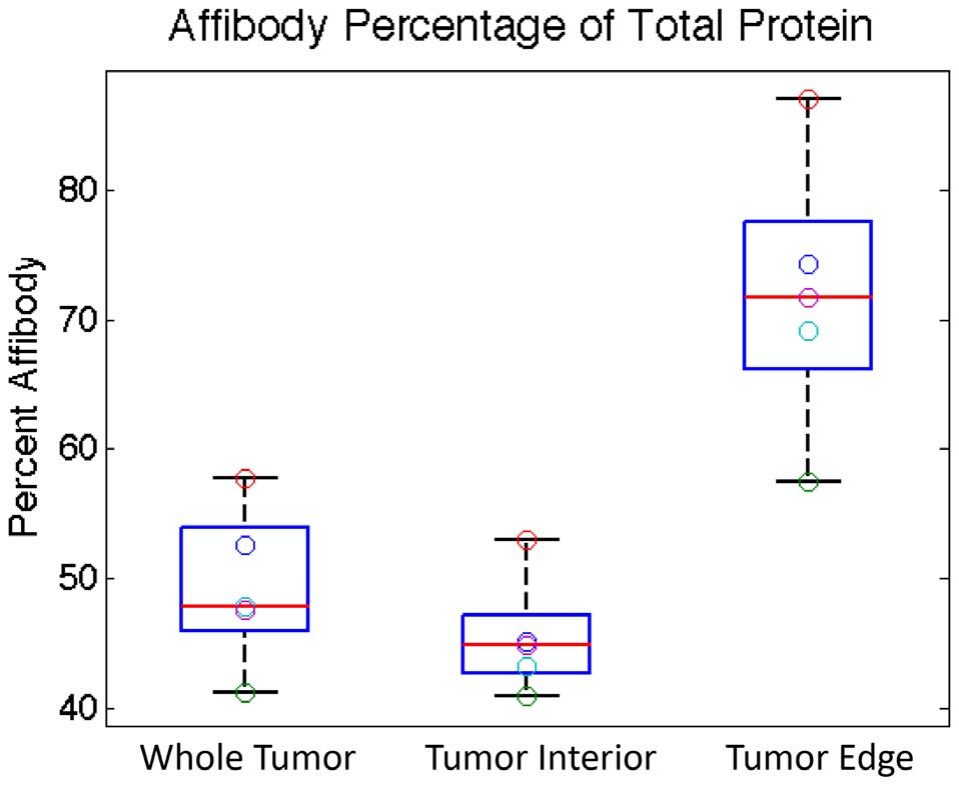
Comparison of protein concentrations in different regions of the tumor. Concentrations are calculated as described in equation (1). The percentage of protein that is Affibody is shown for the whole tumor, tumor interior and tumor edge using box and whisker plots. The central lines are the medians, the edges of the boxes are the 25th and 75th percentiles and individual data points are plotted as open circles.

The conversion from raw signal to estimated protein concentration allowed delivery of the proteins to different regions of the tumor to be compared. In order to quantify this comparison the concentration of Affibody as a percentage of total protein concentration was determined for each region of the tumor. The results can be seen in Fig. 5. While on average the overall delivery of Affibody to the tumor was nearly identical to that of cetuximab, there was a distinct increase in the Affibody protein fraction in the tumor edge compared to the tumor interior. This increase was observed in all animals and ranged from approximately 40% to 65%. On average the percentage of Affibody to total protein in the tumor interior was only 45±5%, while at the tumor edge the fraction of Affibody was 72±11%. (*p = *0.003).

**Figure 6:**
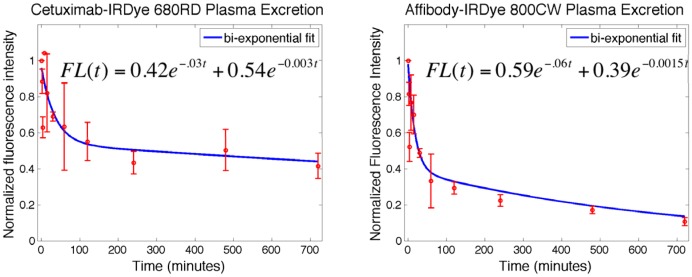
Comparison of plasma excretion for the two proteins. Plasma excretion data with error bars and bi-exponential curve fits to the data are shown for cetuximab-IRDye 680RD (left) and Affibody-IRDye 800CW (right). Curve fit equations are also shown where FL is fluorescence intensity. R-squared values of 0.71 and 0.90 for cetuximab and Affibody fits respectively.

To fully understand the delivery characteristics of both tracers, plasma excretion curves were measured for both cetuximab-IRDye 680RD and anti-EGFR Affibody-IRDye 800CW and a bi-exponential decay equation was fit to the data (Fig. 6). [35], [36] The plasma clearance for cetuximab was found to be significantly slower than for the Affibody with decay constants of 0.03 min^−1^ and 0.0003 min^−1^ for the cetuximab clearance, compared to 0.05 min^−1^ and 0.002 min^−1^ for the Affibody clearance. At the 1 h time point of interest in this study approximately 66±15% of the Affibody present one minute following injection was cleared from the blood, while only 37±24% of the cetuximab present one minute following injection was cleared from the blood.

## Discussion

The injection of fluorescent tracers targeted to molecular receptors over-expressed in tumors, such as EGFR, provide a promising means of improving tumor contrast during surgical resection. The great potential of tumor receptor targeting for both diagnostic and therapeutic applications has led to the development of a number of potential agents that can be used to target specific receptors, all of which can vary greatly in size, lipophilicity, charge, and target affinity. [37]–[39] The choice of the optimal tracer for a specific application is not as simple as just choosing the agent with the highest targeted affinity since many other factors, such as vascular permeability, lymphatic drainage, and plasma clearance, also influence the delivery and retention of targeted imaging agents. In this study, the uptake distribution of two promising EGFR targeted tracers, each having considerably different physical properties were compared in an orthotopic glioma model in athymic mice. The first tracer was a monoclonal antibody, cetuximab, which has a high affinity for EGFR with a K_D_ of 0.1 nM. [40], [41] While this would presumably increase the likelihood of retention, cetuximab is also quite large (152 kDa), [42] which could hinder its ability to perfuse out of the vasculature and into the tumor, especially in areas of only partial BBB breakdown. [21] The second tracer was an anti-EGFR Affibody, which is considerably smaller in size (6.7 kDa), but also has a significantly lower affinity for EGFR with a K_D_ of 2.8 nM. [43], [44] The purpose of this study was to determine which of these tracers would provide inherently better tumor contrast in the context of fluorescence guided resection of gliomas, wherein the integrity of the BBB may play a significant role in regulating the perfusion of the tracers out of the vasculature and into more invasive regions of the tumor margins. [20]–[22] This was carried out by labeling the cetuximab and Affibody with different fluorophores, mixing them in equal protein concentrations, and injecting them simultaneously into mice inoculated with an orthotopic human U251 glioma grown in the left cerebral hemisphere.

On average, nearly equal concentrations of cetuximab and Affibody were measured in the tumors at 1 h post-tracer injection; however, an analysis of the spatial distribution of both proteins demonstrated that there was significantly more Affibody than cetuximab present in the outer edges of the tumor (roughly twice as much Affibody as cetuximab was found in the outer 50 um of the tumor). These are especially interesting results since cetuximab is known to have a 30 times greater affinity for EGFR than anti-EGFR Affibody and was seen to remain in the plasma significantly longer than the Affibody (Fig. 6). Greater time in the blood would result in greater delivery to the tumor if the extravasation characteristics of the two proteins were the same. Additionally, a higher affinity would result in greater tumor retention. It is believed that the nearly equal concentrations within the tumor is the result of the greater permeability of the tumor vasculature to the smaller Affibody than to the larger antibody. In fact, using a two-tissue compartment model [45] to estimate the expected relative uptakes of cetuximab to Affibody based on the measured plasma curves and their theoretical affinities for EGFR, with all other parameters being equal, the cetuximab concentration was predicted to be about 4 times higher than that of the Affibody. The fact that this was not the case suggests that the relative differences in vascular permeability between the two proteins played a significant role in their delivery to the tumor regions, particularly along the tumor edge. It should be noted that the administered doses are low enough that receptor saturation or competitive binding between the Affibody and cetuximab is not expected [46].

The presumed differences in vascular permeability between the Affibody and cetuximab in the tumor interior compared to the tumor edge are in conjunction with expected differences in the extent of the breakdown of the BBB in these areas. Breakdown of the BBB is generally less complete in newly formed regions of the tumor such as the infiltrative edge [20]–[22] and the Affibody protein fraction was most likely higher in these regions owing to the substantially smaller size of the Affibody (∼7 vs. 150 kDa). The Affibody’s superior delivery to the tumor edge is an important finding in the context of fluorescence guided surgical resection as it is the tumor edge that tends to be the most difficult to differentiate from normal tissue and any tracer that better marks these areas has a distinct advantage. [12], [47] Presumably this would also be true for isolated glioma cells; however, this was not investigated in the present study as the orthotopic injection of glioma cells results in limited tumor cell diffusion making it difficult to examine isolated groups of cells. The extent to which the relative increased delivery of Affibody to the tumor edge was the result the Affibody’s greater vascular extravasation in those regions vs. that which was do its presumed higher rate of diffusion was not examined here. However, this is certainly an important question that must be addressed in future studies considering the clinical situation in which distance are such that diffusion may not play a significant role in delivery. [48], [49] While there are numerous factors affecting tracer delivery, the apparent importance of size certainly suggests that it would be worth examining the performance of other small targeted proteins. The most obvious is the natural ligand to EGFR, epidermal growth factor (EGF). EGF is of similar size and affinity to the Affibody [46], [50], however, a primary advantage of using proteins such as the Affibody or cetuximab is that, unlike EGF, they do not activate the EGFR signaling pathway, which could incite tumor growth. [38], [51], [52] In addition to Affibodies, there is a large number of other engineered targeted protein alternatives to full sized antibodies. These include antibody fragments [39] as well as other non-immunoglobulin derived proteins such as DARPIins and Anticalins. [39], [53] Each of these classes of proteins should be considered for their potential use as tracers given the current findings.

The possibility of further modification and optimization of the Affibody used in the present study should also be considered. Affibody plasma clearance could be increased through chemical modification and binding affinity might be enhanced through improved protein engineering designs. Affibody dimers are available as imaging agents with a near two fold improvement in binding affinity due to this bivalency. [43] Despite the fact that dimers would likely have a more difficult time penetrating areas with a more intact BBB, their size is still an order of magnitude below that of antibodies. While the longer plasma half-life of cetuximab allows for greater tumor uptake it also increases background signal due to its greater concentration in the blood at the time of imaging. Any attempts to increase tumor uptake of Affibodies through extending plasma half-life would have to consider this tradeoff. Increased time between injection and imaging may also improve signal-to-background ratios and certainly should be considered moving forward.

### Conclusions

The present study quantifies significant differences in delivery to the margins of orthotopic human glioma xenografts between two fluorescently labeled EGFR targeted proteins. While cetuximab and Affibody had a nearly identical concentrations within the tumor, the concentration of the cetuximab tracer was more confined to the interior of the tumor where BBB breakdown is more complete. The smaller Affibody, with a nearly 30 times lower affinity and a shorter plasma half life, was found in concentrations more than double those of cetuximab in the tumor periphery. The equal or higher abundance of the Affibody compared to cetuximab, particularly along the edges of the tumors was likely a result of the incomplete breakdown of the BBB and the size difference between the two proteins. These results suggest that the size of a targeting agent (the Affibody is only 6.7 kDa compared to cetuximab which is 152 kDa) may be a more important parameter than target affinity when choosing an imaging agent for providing delineation of tumor boundaries during fluorescence guided surgery in neurological oncology. This finding is important for the further investigation and development of fluorescent tracers that are optimized for marking of the tumor periphery. Small, fluorescently labeled proteins with high affinity to tumor receptors show considerable potential for aiding in surgical visualization and the targeted Affibody examined shows excellent potential for EGFR positive tumor targeting.
